# Use of HIV and HSV-2 Biomarkers in Sub-Saharan Adolescent Prevention Research: A Comparison of Two Approaches

**DOI:** 10.1007/s10935-014-0343-6

**Published:** 2014-03-29

**Authors:** Winnie K. Luseno, Denise Dion Hallfors, Hyunsan Cho, Bonita J. Iritani, Joel Adze, Simbarashe Rusakaniko, Isabella Mbai, Benson Milimo, Marcia Hobbs

**Affiliations:** 1 Pacific Institute for Research and Evaluation, 1516 E. Franklin St., Suite 200, Chapel Hill, NC 27514 USA; 2 Department of Maternal and Child Health, Gillings School of Global Public Health, University of North Carolina, Chapel Hill, NC USA; 3 Barau Dikko Specialist Hospital, Kaduna, Nigeria; 4 Department of Community Medicine, University of Zimbabwe, Harare, Zimbabwe; 5 School of Nursing, Moi University, Eldoret, Kenya; 6 Departments of Medicine and Microbiology and Immunology, University of North Carolina, Chapel Hill, NC USA

**Keywords:** HIV, HIV prevention, HSV-2, Adolescent prevention trials, Biomarker data, Sub-Saharan Africa

## Abstract

Self-report of sexual behavior among adolescents is notoriously inconsistent, yet such measures are commonly used as outcomes for human immunodeficiency virus (HIV) prevention intervention trials. There has been a growing interest in the use of HIV and other sexually transmitted disease biomarkers as more valid measures of intervention impact in high HIV prevalence areas, particularly in sub-Saharan Africa. We examine the challenges, benefits, and feasibility of including HIV and herpes simplex virus type 2 (HSV-2) biomarker data, with details about different data collection and disclosure methods from two adolescent prevention trials in Kenya and Zimbabwe. In Kenya, whole blood samples were collected using venipuncture; adult guardians were present during biomarker procedures and test results were disclosed to participants and their guardians. In contrast, in Zimbabwe, samples were collected using finger pricks for dried blood spots (DBS); guardians were not present during biomarker procedures, and results were not disclosed to participants and/or their guardians. In both countries, prevalence in the study samples was low. Although the standard of care for testing for HIV and other sexually transmitted infections includes disclosure in the presence of a guardian for adolescents under age 18, we conclude that more research about the risks and benefits of disclosure to adolescents in the context of a clinical trial is needed. Notably, current serological diagnosis for HSV-2 has a low positive predictive value when prevalence is low, resulting in an unacceptable proportion of false positives and serious concerns about disclosing test results to adolescents within a trial. We also conclude that the DBS approach is more convenient and efficient than venipuncture for field research, although both approaches are feasible. Manufacturer validation studies using DBS for HSV-2, however, are needed for widespread use.

## Introduction

Adolescents in sub-Saharan Africa face high exposure to human immunodeficiency virus (HIV) infection. Despite all efforts to prevent the disease, prevalence estimates in sub-Saharan UNAIDS (i.e., The Joint United Nations Programme on HIV and AIDS) priority countries range from 1.3 to 15.6 % among young women and 0.5–6.5 % among young men aged 15–24 years (UNAIDS, 2011). Higher prevalence estimates are associated with certain regions (e.g., Eastern and Southern Africa) and subpopulations (e.g., adolescent orphaned and/or married girls; Birdthistle et al., 2008; Operario, Underhill, Chuong, & Cluver, 2011; Pascoe et al., 2010). In addition, United Nations Children’s Fund (UNICEF) estimated there were 2 million adolescents aged 10–19 years living with HIV globally in 2009 (UNICEF, 2011). Of these, an estimated 1.2 million were in Eastern and Southern Africa. Kenya and Zimbabwe were among countries with the highest numbers of infected adolescents with an estimated 136,000 (prevalence estimate 1.5 %)^1^ and 104,000 (prevalence estimate 3.1 %) adolescents living with HIV, respectively (UNICEF, 2011). Thus, effective HIV prevention interventions for young people are greatly needed.

The true measure of effectiveness for such interventions is reduction in HIV and other sexually transmitted infections (STIs). However, very few adolescent prevention studies have used biomarker data as outcomes (Michielsen et al., 2010) because of concerns about the difficulties in collecting these data in resource poor settings (Jukes, Simmons, & Bundy, 2008), even when power analyses suggest that moderate intervention effects could be detected. Behavioral prevention scientists have instead relied on self-reported sexual behavior, which is subject to social desirability bias and likely to differ between study arms (Cowan & Pettifor, 2009; Kirby, Obasi, & Laris, 2006). Widespread access to testing and treatment facilitated by the President’s Emergency Plan for AIDS Relief (PEPFAR; http://www.usaid.gov/what-we-do/global-health/hiv-and-aids) have greatly enhanced opportunities to successfully incorporate biomarker procedures into adolescent HIV prevention research designs, since infected individuals identified through testing offered by studies may access free or subsidized HIV treatment and care (Stringer et al., 2006; Wools-Kaloustian et al., 2006, 2009). Moreover, including biomarker outcomes can greatly improve the credibility and legitimacy of HIV prevention research.

To address the issue of feasibility, we present two case studies of prevention trials with biomarker data collection among adolescents in Kenya and Zimbabwe. Together these studies provide a valuable opportunity to examine the ethical and practical challenges of biomarker procedures within HIV prevention intervention clinical trials in developing countries. Specifically, we describe the challenges faced by investigators in the two cases in developing and implementing ethical procedures for informed consent, biomarker testing, and disclosure of test results. We also present the practical challenges of collecting biological samples in resource-limited countries and compare sample collection by blood spots relative to venipuncture. In addition, we examine difficulties with analyzing biological samples when the performance of assays in certain populations is unclear. We also provide specific suggestions for further research and development in collecting and analyzing biomarker data for adolescent prevention research.

The primary goal of HIV prevention among adolescents (ages 10–19 years) is to reduce new infections and related sexual risk factors [World Health Organization (WHO), 2006). Although the HIV prevalence of adolescents ages 15 and older is monitored through Demographic and Health Surveys (DHS)^2^ in most sub-Saharan Africa countries, little is known about the prevalence of HIV in adolescents under 15 years. Moreover, these adolescents are rarely tested for HIV in clinical settings unless they present in poor health or with recurrent infections (Ferrand et al., 2009).

Although a small number of recent randomized controlled trials have used biomarkers (Baird, McIntosh, & Ozler, 2010; Cowan et al., 2008; Jewkes et al., 2006; Pronyk et al., 2006; Ross et al., 2007), most adolescent HIV prevention studies have relied on changes in self-reported sexual behavior to assess intervention effectiveness (see reviews: Cowan & Pettifor, 2009; Michielsen et al., 2010), with audio computer-assisted self-interviews intended to improve veracity (Mensch, Erulkar, & Hewett, 2003). However, biomarker comparisons have suggested that self-reported measures have low validity (Cowan et al., 2002; Gavin et al., 2006; Mensch, Hewett, Gregory, & Helleringer, 2008; Palen et al., 2008; Plummer et al., 2004). For example, in one study, only one of 16 youth with HIV admitted to ever having sex, and four of the 16 also had either chlamydia or gonorrhea (Cowan et al., 2002). Inclusion of a highly prevalent and reliable biomarker of sexual activity in conjunction with self-reported sexual behavior can help to improve reliability of this outcome measure. HIV infection is not the most useful biomarker of adolescent sexual activity by itself because of the relatively low prevalence among younger adolescents and the potential for transmission through perinatal and non-sexual blood-borne routes (Amornkul et al., 2009; Ferrand et al., 2009; Stover, Walker, Grassly, & Marston, 2006).

Herpes simplex virus type 2 (HSV-2), the primary cause of genital herpes, is a commonly used biomarker of sexual activity because the presence of HSV-2 antibodies is highly associated with past sexual behavior (Tobian et al., 2009; Van de Laar et al., 1998). In contrast to other STIs such as chlamydia, gonorrhea, syphilis and trichomoniasis, infection with HSV-2 is life-long, and, once established, there is currently no treatment to eliminate it (Geretti, 2006; Gupta, Warren, & Wald, 2008). HSV-2 prevalence in the adult general population in sub-Saharan Africa is high, ranging from 30 to 80 % in women and from 10 to 50 % in men, as assessed by detection of HSV-2 antibodies [Amornkul et al., 2009; Ghebremichael, Larsen, & Paintsil, 2009; Kjetland et al., 2005; Munjoma et al., 2010; National AIDS & STI Control Programme (NASCOP), Republic of Kenya, 2009; Tobian et al., 2009; Weiss, 2004]. Prevalence estimates for adolescents are only available through community-based studies and are lower. One study in Zimbabwe among adolescent females aged 15–19 years reported that 12 % of participants tested positive for HSV-2 (Birdthistle et al., 2008). A study in western Kenya found HSV-2 prevalence to be 9 % for females and 4 % for males aged 13–14, and 28 % for females and 17 % for males aged 15–19 years (Amornkul et al., 2009). Another study conducted in Zimbabwe among adolescents whose mean age was 15 years reported a prevalence estimate of 0.2 %, suggesting a later stage of sexual debut (Cowan et al., 2008).

Bastien et al. (2012) evaluated the use of HSV-2 as a biomarker of sexual debut among young people aged 10–24 years. Following a comprehensive review of the literature, the authors concluded that the use of HSV-2 as a biomarker for sexual debut is limited because of its low transmissibility and the fact that not all potential sexual partners are infected with the virus. Building on these findings, our study describes the challenges of collecting HIV and HSV-2 biological data as biomarkers for sexual activity among sub-Saharan African youth in prevention trials.

## Brief Description of Case Studies

We present two clinical trial case studies to examine important issues related to adolescent biomarker data collection in two clinical trials in Kenya and Zimbabwe (Hallfors et al., 2011). Both studies were school-based, cluster randomized trials testing school support (including payment of school tuition and school uniforms) as a structural HIV prevention intervention for adolescent orphans. In both studies, we collected blood samples from participants for HIV and HSV-2 testing, but they differed as follows: we collected samples by venipuncture with results disclosed to participants and their guardians in Kenya, whereas in Zimbabwe, we collected samples by finger pricks for dried blood spots (DBS) and results were not disclosed. The Kenya sample (*N* = 837) comprised young adolescent male (52 %) and female (48 %) orphans in Grades 7 (61 %) and 8 (39 %) enrolled in school in Siaya District, Nyanza Province. Their mean age was 14.9 (*SD* 1.5; range 11–21 years); 22 % reported ever having had sex. Among girls, 1 % had ever been pregnant. None of the boys reported ever fathering a child. Specimens were collected at baseline in October 2011. HIV and HSV-2 prevalence was 1 % (*n* = 10) and 3 % (*n* = 28), respectively. Among those with positive HIV or HSV-2 test results, 70 % (*n* = 7) and 64 % (*n* = 18), respectively, reported never having had sex.

The original sample for the Zimbabwe study was orphan 6th grade girls (*N* = 328) in Manicaland Province primary schools; surveys were administered annually during 2007–2010 (Hallfors et al., 2011). After securing a project renewal grant, we collected biomarker specimens and a final survey in March through June 2012, when most participants were 16–17 years old. From the original sample, we located 287 (88 %) for the 2012 survey. Of these, 79 % were still enrolled in school. Twenty-three percent reported ever having sex; 18 % had ever been married and/or pregnant. HIV and HSV-2 prevalence was 4 % (*n* = 12) and 6 % (*n* = 16), respectively. Among those with positive HIV or HSV-2 test results, 50 % (*n* = 6) and 44 % (*n* = 7), respectively, reported never having had sex.

## Ethical Considerations Including Consenting Procedures

Although the protection of adult participants in HIV prevention research is well developed (National Institute of Mental Health Collaborative HIV/STD Prevention Trial Group, 2007), we found that clear guidance regarding HIV and other STI testing and disclosure is lacking for children and adolescents. As an example, our two clinical trials were peer-reviewed by two different NIH scientific review panels; one set of reviewers thought that it was an ethical imperative to disclose HSV-2 as well as HIV results to adolescents, whereas the other was concerned that disclosure of biomarker results might lead to stigma and abuse. These divergent opinions led to discrepant protocols for disclosure, with one trial disclosing and the other not disclosing test results to participants.

We used the National HIV testing and counseling (HTC) guidelines [Central Statistical Office (CSO) & Macro International Inc., 2007; NASCOP, Republic of Kenya, 2008] for HIV in both countries. Given the lack of national guidelines for adolescent STI testing in Kenya [Ministry of Health (MoH), Republic of Kenya, 2006), we developed a protocol in conjunction with the Siaya District AIDS and STD Control Office that closely followed HIV guidelines. Study protocols were reviewed and approved by IRBs in the US, Kenya and Zimbabwe.

The Kenya study was approved by the Ministry of Education and Siaya District Education Office; school head teachers identified orphans and organized meetings in which Moi University research collaborators and a District Education Officer explained the study to students and their caregivers. Caregivers and students were provided the opportunity to ask questions about the study and biomarker data collection. Both the caregiver and the orphan signed the consent form. Study participation was voluntary but required consent to biomarker testing. We invited a total of 923 orphans in grades 7 and 8 in 2011 to participate; 849 (92 %) consented and 837 (91 %) completed both the student survey and biomarker testing.

Following national guidelines, we required Kenya study participants under age 18 to have an adult guardian accompany them for HIV rapid testing (NASCOP, Republic of Kenya, 2008). In some instances, however, the guardian was not available because of work, other commitments, or illness. In such cases we permitted a relative, teacher or neighbor to stand-in for the guardian if they had a note from the guardian or if research staff were able to speak with the guardian by phone. Given the sensitive nature of HIV testing, the senior counselor interviewed all unclear or undocumented cases to determine whether or not to proceed with specimen collection and HTC. We visited clinics several times to give participants the opportunity to return for testing with their guardian or authorized proxy. Although not required, all participants ages 18 and older were accompanied by an adult for support in case of a positive HIV test result. In accordance with national guidelines, we gave referrals to publicly funded HIV care and treatment centers to all adolescents with positive HIV test results, while HIV negative participants were counseled on risk reduction (NASCOP, Republic of Kenya, 2008). We disclosed results to both guardians and participants if the latter were under age 18 and to the participants alone if they were 18 years old according to the study protocol.

In Zimbabwe, with permission from the Provincial Education Officer, the consortium Principal Investigator and a Research Associate held meetings at study schools to explain the continuing study to parents/guardians, participants, and key school staff. We obtained written parent/guardian consent and participant assent for continued voluntary participation in the study, including separate checkbox consent for survey and blood sample collection. Guardians raised few concerns, although they did ask why we were not disclosing test results.

Participants who were married or 18 years or older provided their own consent. In deference to local cultural norms and to prevent potential spousal abuse, we prepared a second consent form for husbands to provide consent for their wives’ participation. Most of the wives gave their own consent for the survey and did not ask their husbands for consent. In four cases, the husband signed a consent form and in five additional cases, the husband refused and the wife did not take the survey. Although asking husbands for permission for their wives’ study participation is not consistent with US culture, it appears to be a useful protocol accommodation for some young wives in rural Zimbabwe. We consistently relied on local researchers and key informants to make such culturally sensitive decisions.

After 4 months, we found 88 % of the original sample who consented to the survey; 85 % consented to blood specimen collection. Of those not surveyed, two participants had died in childbirth, 2 % refused, and approximately 10 % of the sample could not be found. Of those not consenting to blood specimen collection (*n* = 8), six were married and refused for religious reasons or husband’s objection. Two were consented for the survey by school officials who refused to provide additional consent for the blood collection, and no guardian was available to give permission.

## Collecting and Analyzing Biological Samples

HIV testing is technically far more advanced for widespread use in sub-Saharan Africa than HSV-2 testing. Rapid HIV antibody tests are now widely used and accepted (MoH, Republic of Kenya, 2006), and sequential algorithms for retesting have been adopted in Kenya and Zimbabwe for rapid testing (CSO & Macro International Inc., 2007; NASCOP, Republic of Kenya, 2008; WHO, 2004). Rapid HIV tests meet high performance standards, sample collection procedures are simple, tests are easy to perform, and interpretation of test results is reasonably clear.

In contrast, HSV-2 serologic testing presents some important challenges. Although a number of HSV-2 type-specific serologic tests are available, only a limited number have been tested among sub-Saharan African populations. These include the HerpeSelect HSV-2 enzyme-linked immunosorbent assay (ELISA; Focus Diagnostics, Cypress, CA) and Kalon HSV-2 ELISA (Kalon Biological Ltd., Guildford, UK; Biraro, Mayaud, Morrow, Grosskurth, & Weiss, 2011). These tests require skilled laboratory staff, specialized equipment, and a constant source of electricity. They are appropriate for use with large batches of samples as in sentinel surveys and are inexpensive, costing about $3 per test.

Although the literature reports variable performance of these tests with respect to sensitivity and specificity in sub-Saharan populations, Kalon is often found to be superior to Focus HerpeSelect (see Biraro et al., 2011 for a review of the performance of HSV-2 tests in sub-Saharan Africa; Delany-Moretlwe et al., 2010; Gamiel et al., 2008; Ng’ayo, Friedrich, Holmes, Bukusi, & Morrow, 2010; Smith et al., 2009; van Dyck et al., 2004). When using the manufacturer’s instructions (index cutoff value = 1.1 for both tests), Kalon tends to have lower sensitivity but higher specificity than Focus HerpeSelect (Delany-Moretlwe et al., 2010; Gamiel et al., 2008; Ng’ayo et al., 2010; Smith et al., 2009; van Dyck et al., 2004). The low specificity of Focus HerpeSelect is particularly problematic when disclosing results to research participants because of an unacceptably high rate of false positives when prevalence is low (Gamiel et al., 2008; Mark et al., 2008; Ng’ayo et al., 2010; van Dyck et al., 2004).

Increasing the assay cut-off values is one strategy for improving the performance of both the Kalon and Focus HerpeSelect tests. A number of studies have shown that increasing the cut-off value for Focus HerpeSelect to 3.5 increases specificity, although sensitivity is necessarily reduced (Delany-Moretlwe et al., 2010; Smith et al., 2009). Other studies similarly improved performance at cut-off values of 3.2 and 3.3 (Delany-Moretlwe et al., 2010; Gamiel et al., 2008; Ng’ayo et al., 2010). Although findings are mixed, increasing the cut-off value for the Kalon test to 1.5 has also been shown to increase specificity, with an attendant reduction in sensitivity (Gamiel et al., 2008; Ng’ayo et al., 2010; Smith et al., 2009).

In our studies, the Kenya laboratory used the Kalon assay, and the Zimbabwe laboratory used the Focus test. At the time of our studies, neither assay had been validated for DBS by the manufacturer in African populations. The efficiency of DBS for HSV-2 testing with each assay must be evaluated in specific populations prior to use (Hogrefe, Ernst, & Su, 2002). Because the Kalon test had not been validated for HSV-2 testing with DBS samples, specimens in the Kenya study were obtained by venipuncture. The Zimbabwe laboratory had validated the Focus test with DBS (Mudzori & Mutsogoro, 2006). In both studies, we used a modified algorithm to interpret results: samples with initial index values <0.9 were reported as negative and no further testing was conducted. Samples with initial index values >2.5 were defined as high positive and were reported as positive without additional testing. Samples with initial index values from 1.5 to 2.5 were considered low positive, and those with initial index values between 0.9 and 1.5 were considered equivocal; all low positives and equivocals were retested in duplicate. If both retest results had index values >1.5, the final result was reported as positive. If both retest results were <0.9, the final result was negative. If retest results were equivocal (0.9–1.5) or discordant, the final result was indeterminate.

Although test performance was improved by increasing the cut-off and expanding the range of results for repeat testing, the performance characteristics of tests to detect antibodies to HSV-2 are imperfect. Based on a recent systematic review of HSV-2 antibody test performance in Sub-Saharan Africa (Biraro et al., 2011), sensitivity for the Kalon test used in Kenya was estimated at 94 % and specificity at 92 % with the higher cut-off value of 1.5. The predictive values of all diagnostic tests are strongly influenced by the prevalence of infection in the population, and in low prevalence situations, even a very good test will have a poor positive predictive value (PPV). Using the sensitivity and specificity values for the Kalon test under the testing conditions employed in our Kenya study, the PPV was estimated at 26 % with a 3 % HSV-2 prevalence. That is, we can be confident that about one in four participants with positive test results truly had HSV-2 antibodies present in their blood sample; however, as many as three in four could have had a false positive test result.

## Feasibility Comparison of Sample Collection Methods

In Kenya, we conducted sample collection and HTC primarily in local public health facilities near the schools. Eighteen facilities participated. In two sites, with permission from the school headmasters and district education officer, we collected blood samples and conducted HTC at the school. All services were provided at no cost to participants.

Venipuncture was conducted by six skilled phlebotomists trained in HTC. To avoid obtaining two separate blood specimens (finger prick for rapid HIV testing and venipuncture for HSV-2 testing), we used venous blood samples for both tests. The process, including counseling, blood draw, HIV testing, disclosure of results took on average 20 min. The process was longer for participants with discordant results who needed a tie-breaker test and for participants with HIV positive results. We offered all participants and their guardians a snack either before or after specimen collection.

We stored blood samples for HSV-2 testing in labeled vacutainer tubes in a cooler box without ice and transported them to a local laboratory within 24 h for serum processing. Sera were stored at the local laboratory in a freezer at −20 °C until transport on ice weekly to the HSV-2 testing laboratory. The logistics of specimen collection, processing and transport for this study were challenging but feasible.

In comparison, Zimbabwe DBS data collection was much less challenging. We did not require guardians to be present because HIV results were not provided to participants. Finger pricks were done quickly, taking only 3 min. Sampling and DBS preparation were much less obvious to others nearby, helping to safeguard participant privacy and reduce concerns about witchcraft or other conspiracy theories regarding HIV/AIDS and blood, which circulate in Sub-Saharan African communities (Tenkorang, Gyimah, Maticka-Tyndale, & Adjei, 2011).

Blood from the finger prick was dropped on five circles on a DBS filter card labeled with the participant’s study ID. Some participants may have been dehydrated or not very well nourished, making it difficult to fill all circles, but adequate samples were collected from all participants. Specimens were dried, stored with a desiccant, and transported to the testing lab at ambient temperatures. DBS samples are easy to store and transport and require no processing or refrigeration (see Table 1 for a summary of the study procedures).

**Table 1:** Comparison of biomarker testing procedures in two research studies

	Kenya	Zimbabwe
Ethical considerations	Written guardian consent if <18/adolescent assent	Written guardian consent/adolescent assent
	Results disclosed to guardian and adolescent	No results disclosed
	Referrals for care	Not applicable
Collecting and analyzing biological samples		
Method	Venipuncture	Dried blood spot
HIV test	Rapid test	Rapid test
HSV test	Kalon ELISA	Focus ELISA
Feasibility of collection methods		
Time	20 min	3 min
Temperature for specimen transport	Cooler with no ice	Ambient
Testing costs	Reference lab	Reference lab
	Bench fee, supplies, storage, labor US$ 5,784	Bench fee, supplies, labor US$ 8,000
	Field site lab	
	Sample processing, labor, supplies, storage US$ 3,611	Additional fee US$ 480
	Other supplies US$ 100	
	Transportation US$ 375	
	Total US$ 9,919	Total US$ 8,480

*ELISA* enzyme-linked immunosorbent assay

## Discussion

Given the experience we have described, we conclude that it is feasible to obtain HIV and HSV-2 biomarker data for adolescent HIV prevention intervention studies. In particular, the use of HIV data is quite well-developed. The US PEPFAR program has accomplished considerable staff training in sub-Saharan Africa; it has also stimulated the development of well-equipped laboratories, as well as useful tools for testing and protocols for improving test sensitivity, specificity, and cost (Stringer et al., 2006). In addition, it has stimulated the development of national guidelines (particularly for adults) for the disclosure of results. For HIV positive persons, free ARV treatment is available according to well-defined protocols. However, HSV-2 testing has not experienced a similar investment. As a result, incorporation of HSV-2 serology results poses considerable challenges, particularly if results are disclosed to participants.

Based on our experience, there are two reasons why we do not recommend disclosing HSV-2 serology results to adolescent human subjects in sub-Saharan Africa. First, in populations with low or moderate prevalence of infection, as might be expected for adolescents, the potential for false positive test results is substantial in combination with relatively minor imperfections in test specificity. HSV-2 infection is often a silent disease, insofar as only 10–25 % of people with antibodies for the condition are aware that they have genital herpes (Fleming et al., 1997; Leone, Fleming, Gilsenan, Li, & Justus, 2004; Sizemore, Lakeman, Whitley, Hughes, & Hook, 2006). Adolescents without symptoms may deny ever having had sexual intercourse^3^, further complicating the clinical picture and raising concerns about false positives with the potential for psychological and social harm related to participation in the study. Second, local departments of health and STI authorities in sub-Saharan African countries were much less familiar with HSV-2 pathology and treatment than with HIV and some other STIs. Even in cases where subjects acknowledge sexual exposure or genital herpes symptoms, local health clinics may not be prepared to provide appropriate treatment or care. Given these vexing problems, the wisdom of disclosing HSV-2 results to adolescents after testing within a clinical prevention trial is questionable. In particular, decisions about whether to inform adolescent participants in prevention trials about HSV-2 test results should be made only after prevalence data are available and the predictive values of the test can be accurately assessed.

In light of the feedback from the NIH study section that raised concerns about possible stigma and abuse of HIV-positive participants in Zimbabwe, as well as our own concerns, we recommend that more research be conducted to study the consequences of disclosure to adolescents in sub-Saharan Africa. A review of the literature indicates that sub-Saharan adolescent prevention studies with biomarker outcomes have taken a variety of approaches to disclosure. In some studies, counselors disclosed participants’ HIV results immediately after rapid testing (Baird, Garfein, McIntosh, & Ozler, 2012; Dalal et al., 2012; Medley et al., 2012). In other studies, participants could choose to receive their results after testing, either immediately or a few weeks later (Amornkul et al., 2009; Jewkes et al., 2006; Ross et al., 2007). In still other studies, the research did not provide test results but voluntary counseling and testing (VCT) was made freely available for study participants (Birdthistle et al., 2008; Cowan et al., 2002, 2008). None of these studies, however, examined whether adolescents experienced any untoward outcomes with testing and disclosure, whether HIV positive youth enrolled in care or took steps to prevent transmission, whether HIV negative youth benefited from prevention counseling and engaged in subsequent testing, or if adolescents and their guardians felt it was appropriate to learn their status in the context of a research study.

From our Kenya study, the problem of stigma did not appear to be a major obstacle to adolescent testing and disclosure in our study area. Perhaps this is because HIV prevalence is among the highest in the country, and few families have been unaffected by HIV/AIDS. Moreover, high HIV prevalence has drawn research, education, and HIV counseling and testing campaigns to the area. For example, in our Kenya clinical trial, more than 50 % of participants (all in grade 7 and 8) reported that they had previously been tested for HIV, mostly through a home-based testing initiative of the Kenya Medical Research Institute (KEMRI) and US Centers for Disease Control and Prevention (CDC). Although our study provided HIV test results to youth, we relied on phlebotomist/nurse counselors to refer youth and their guardians for care, following national guidelines. The onus of making contact with the clinic was left up to the individual and was not tracked by the system of care; and follow up to make sure that HIV positive youth accessed care was well beyond the scope of our study. Since it is unknown whether youth experience significant mental trauma and social prejudice, and whether they actually engage in the system of care, we recommend that further research be conducted to examine the perceptions and behaviors of youth—and particularly newly diagnosed youth—after disclosure of their test results to ascertain the consequences of this aspect of research participation and whether it can be improved. This is particularly important for adolescents, since results are disclosed in the presence of their guardian.

Regarding procedures for collecting biomarkers, we conclude that finger-sticks and DBS are overwhelmingly superior to venipuncture in sample collection efficiency and reduced burden on participants and community institutions. Manufacturer validations of DBS for HSV-2 are urgently needed, however, for this procedure to be more widely accessible to researchers. Because blood collection for DBS is minimally invasive, this procedure has excellent potential for widespread acceptability and consequent high participation in school and community research sites. In addition, rapid HIV testing in sub-Saharan Africa is typically conducted by finger prick, making this the preferred way for trained African health workers and counselors to participate in research data collection (WHO, 2004).

Given the complex decisions required for biomarkers, we recommend that behavioral prevention scientists interested in using biomarker data collaborate from the early planning stages with biomedical scientists who have expert knowledge in HSV-2, as well as HIV, laboratory tests and testing procedures. We found that team members with such expertise provide valuable assistance in selecting qualified in-country laboratories, developing appropriate budgets, protocols, quality assurance and algorithms for interpreting results, and helping research staff to bridge communication with in-country laboratory staff.

Further, we recommend that HSV-2 test kit manufacturers determine optimal cut-off standards for sub-Saharan populations, and that researchers who conduct HSV-2 testing explicitly define the testing cutoffs they use in publications. There is now a substantial literature documenting validity problems when using manufacturers’ cutoffs with African populations potentially inflating prevalence findings (Gamiel et al., 2008; Ng’ayo et al., 2010; van Dyck et al., 2004). This suggests the need for action to improve assay validation practices on the part of manufacturers as well as methodological details from researchers documenting their findings.

When conducting collaborative international research, it is important to recognize that alternative ethical systems exist. These systems are shaped by local cultural values and norms. Thus, for international research we recommend inclusion of team members who are knowledgeable about local contexts. Indeed, successful collaborative research partnerships respect local cultures, values and practices; negotiate effectively within these systems; and incorporate into study designs ethical practices that are appropriate and sensitive to local cultural contexts (e.g., in our case, providing for husband consent for married participants; Christakis, 1992; Emanuel, Wendler, Killen, & Grady, 2004).

Behavioral prevention scientists traditionally have relied on self-reported sexual behavior survey item measures to evaluate adolescent HIV prevention interventions. Our experiences conducting research with orphan adolescents in Kenya and Zimbabwe suggest that collection of HIV and STI test results even in rural, resource-poor settings in sub-Saharan Africa is a feasible addition to the behavioral research toolkit. The use of STI biomarkers can greatly improve the validity of findings from adolescent behavioral intervention trials. Research is urgently needed to examine the risks and benefits of HIV testing and disclosure of test results in the context of a research study for adolescents. Further development and implementation of STI biomarker assessment techniques—particularly pertaining to using DBS—is needed to advance HIV prevention science.

## Footnotes

1 Prevalence estimates provided here are calculated as the number of infected adolescents aged 10–19 years as a proportion of total number of adolescents aged 10–19 years.

2 Since 1984, DHS surveys have collected and disseminated information on fertility, family planning, maternal and child health, gender, HIV/AIDS, malaria and nutrition. The surveys, which are funded by the United States Agency for International Development (USAID), have been conducted in over 90 countries. For more information about DHS visit http://www.measuredhs.com/.

3 HIV-infected adolescents who deny having had sexual intercourse may have been vertically infected. Further, HIV testing, treatment and care is far more advanced than for HSV-2.
